# Composite Ionogel Electrodes for Polymeric Solid-State Li-Ion Batteries

**DOI:** 10.3390/polym16131763

**Published:** 2024-06-21

**Authors:** Noah B. Schorr, Austin Bhandarkar, Josefine D. McBrayer, A. Alec Talin

**Affiliations:** 1 Department of Power Sources R&D, Sandia National Laboratories, Albuquerque, NM 87123, USA; 2 Department of Material Physics, Sandia National Laboratories, Livermore, CA 94550, USA

**Keywords:** solid-state electrolyte, ionogel, polymer electrolyte, Li-ion battery

## Abstract

Realizing rechargeable cells with practical energy and power density requires electrodes with high active material loading, a remaining challenge for solid-state batteries. Here, we present a new strategy based on ionogel-derived solid-state electrolytes (SSEs) to form composite electrodes that enable high active material loading (>10 mg/cm^2^, ~9 mA/cm^2^ at 1C) in a scalable approach for fabricating Li-ion cells. By tuning the precursor and active materials composition incorporated into the composite lithium titanate electrodes, we achieve near-theoretical capacity utilization at C/5 rates and cells capable of stable cycling at 5.85 mA/cm^2^ (11.70 A/g) with over 99% average Coulombic efficiency at room temperature. Finally, we demonstrate a complete polymeric solid-state cell with a composite anode and a composite lithium iron phosphate cathode with ionogel SSEs, which is capable of stable cycling at a 1C rate.

## 1. Introduction

Despite tremendous progress in the development of solid-state electrolytes (SSEs) with conductivities that rival those of liquid organic electrolytes, realizing practical solid-state batteries (SSBs) with power, capacity, and fabrication costs competitive with those of conventional Li-ion batteries remains a major challenge for the battery industry. Indeed, numerous reports claim superior performance for thin-film SSBs [1,2,3]. Unfortunately, sluggish Li-ion diffusion in the active material and the relatively high cost of manufacturing thin-film SSBs make this approach impractical for most consumer applications such as transportation or large-scale stationary grid energy storage. In contrast, conventional Li-ion batteries achieve high power and energy densities by relying on relatively thick multicomponent electrodes made up of micron to sub-micron size grains of active material mixed with a binder and conductive carbon and a liquid electrolyte that uniformly penetrates and contacts the available active electrode grains [4,5]. Because the electrolyte is a fluid, it is able to form intimate, mechanically compliant interfaces with the tortuous, complex-shaped structure of the cast electrode [6,7]. As a strategy to combine the benefits of liquid electrolytes with those of SSEs, we and a number of other groups have been investigating ionogel (IG)-based electrolytes. IGs consist of an ionic liquid electrolyte (ILE) contained within a solid matrix, where the coexistence of solid and liquid phases combines the mechanical properties of a solid (e.g., structural support and elasticity) with the physical properties of a liquid electrolyte. ILEs are attractive because they have high ionic conductivities, are stable over a broad range of temperatures and chemical environments, are non-flammable, and typically have high voltage windows [8,9,10,11,12]. Furthermore, once solidified, we recently demonstrated that the IG functions as a separator without additional separator membranes [9]. The merits of the varieties of other SSEs have been examined previously and thus will not be discussed in detail [13,14]. One reason polymer-based SSEs are appealing is they can be designed to be flexible [15,16,17], whereas rigid separators such as garnets, oxides, and sulfides are harder to incorporate in many cell formats (e.g., prismatic, cylindrical), limiting the energy density of the battery build. There is a growing field of multicomponent SSEs that incorporate polymers and inorganic Li^+^ conductors that may overcome the drawbacks of purely inorganic separators [18,19,20]. This approach may also be suitable for the future development of IG electrolytes. The fact that IG is deposited as a liquid implies that, in principle, the electrolyte should uniformly penetrate the pores of thick, composite electrodes.

Often, studies of SSBs with IG electrolytes have been performed on electrodes with relatively low loading, typically <5 mg/cm^2^ of active material, well below the ~10 mg/cm^2^ needed for practical applications. To address this issue of low loading, here, we examine the performance of half cells and full batteries with LiFePO_4_ (LFP) and Li_4_Ti_5_O_12_ (LTO) active materials with target loadings of 10 mg/cm^2^. LTO is an ideal candidate to investigate for solid-state batteries because the material is known as a safer alternative to graphite or Li anodes and has been demonstrated to have high rate capabilities, long cycle life, and low strain during (de)intercalation [21,22,23]. The authors also hypothesized that the lessons learned in making LTO composite electrodes could be relevant for the future development of other oxide active materials used in composite electrodes. While LTO has a lower energy density than Li metal, lithium titanate does not have issues with Li dendrite formation during cycling. Therefore, the research is geared toward enabling high-loading intercalation materials instead of enabling conversion reactions (e.g., plating/stripping reactions). In particular, we explore two different fabrication strategies resulting in drastically different charge/discharge behaviors at room temperature. First, we study half cells where the IG is deposited over the cast electrode that includes the active materials plus the binder and conductive carbon. In the second approach, we mix the IG precursors with the active material and the conducting carbon, thus creating a composite electrode where the IG also serves as the binder. We find that the performance for the composite IG half cells is dramatically improved compared to the performance of those formed by the first approach, enabling stable cycling at current densities approaching 6 mA/cm^2^ for single-layer cells. This approach is then applied to making a full LFP||LTO SSB. Our results define a successful strategy for realizing SSBs that combine the benefits of liquid electrolytes with added safety, higher energy density, and custom shape compatibility for SSBs.

## 2. Experimental

The ionogel was synthesized by dissolving poly(vinylidene fluoride co-hexa fluoropropylene) (PVDF-HFP) in acetone (76 g/L). A solution of 1 M lithium bis(fluorosulfonyl)imide (LiFSI) in 1-Butyl-1-methylpyrrolidinium bis(trifluoromethylsulfonyl)imide (PYR14TFSI) was then made. These two solutions were mixed in a 329:53 ratio and vortexed for 10–20 s using a Fisher Scientific Vortex Mixer (Waltham, MA, USA). This ionogel was then used for both the solid-state electrolyte and inclusion into the composite electrodes. PVDF-HFP and acetone were purchased from Sigma Aldrich (St. Louis, MO, USA). LiFSI was purchased from TCI Chemicals (Portland, OR, USA). PYR14TFSI was purchased from Solvionic (Toulouse, France).

The composite cathodes and anodes were produced using either carbon-coated lithium titanate or carbon-coated lithium iron phosphate received from NEI corporation (Somerset, NJ, USA). Precise masses of the active material were mixed at an 8:1 ratio with Danka conductive carbon. Yttria stabilized zirconia micro-milling media was added to the powder mixture and then vortexed for 10 min to ensure homogeneity. The micro-milling media was removed, and premade ionogel was added to form a slurry in volumes dependent on prior calculations. The slurry was vortexed until well mixed.

A total of 225 uL of slurry was drop cast onto a carbon-coated aluminum foil disk and allowed to dry at room temperature until complete acetone evaporation or for at least 1 h. The disks were baked at 100 °C for 3 h. A separator layer of 65 uL of non-slurry ionogel was applied to the electrode materials, which were dried and baked under the same conditions, resulting in a roughly 20 µm film on the surface.

The prepared composite disks were transferred to an inert argon glovebox (MBraun Labmaster II) for all further preparation. A 1 M lithium perchlorate (LiClO_4_) in propylene carbonate (PC) solution was prepared and used for a solvent exchange process. Further details of this procedure are found in previous publications [8,9,17], which found improved capacity retention during room temperature cycling after performing a solvent exchange. Briefly, in this process, the disks with the composite electrodes and ionogel layer were immersed in small volumes of stock solvent exchange solution. The electrodes were soaked in this solution for 3 h, and then the same electrodes were emersed for another 3 h in a fresh solution and then another 2 h in a fresh solution.

Immediately upon completion of the final solvent exchange step, the disks were loaded into the coin cells without any subsequent washing. Coin cell assembly order, from bottom to top, includes the coin cell casing bottom, two 0.5 mm spacers, anodic material (lithium foil or composite LTO), 5 uL of 1 M LiClO_4_ in PC for adhering materials, cathodic material (composite LFP), two 0.2 mm spacers, a wave spring, and the coin cell casing top. Coin cell casing tops and bottoms, spacers, and springs were all purchased from MTI Corp and made of stainless steel. For half cells, glass fiber separators were used with an electrolyte to wet the Li metal anode. Lithium foil was purchased from Sigma Aldrich and was ground against gritted polymer blocks to remove any built-up surface contaminants or reactive byproduct layers. These components were crimped using a MTI Compact Digital Pressure Controlled Electric Crimper MSK-160-E (Hefei, China) at 0.75 tons of force to seal the coin cell casing.

A Biologic VSP-300 (Knoxville, TN, USA) was used to characterize the coin cells by performing galvanostatic cycling with potential limitation. LFP vs. LTO cells were cycled from 0.5 V to 3.0 V, and LTO vs. Li cells were cycled from 1.0 V to 2.5 V.

Scanning electron microscopy (SEM) with energy dispersive spectra (EDS) was performed on the cross-sections of selected electrodes to understand the composition and thickness and as confirmation of ion exchange. Cross-sectioning was performed by cutting the electrode perpendicularly with a sharp blade. SEM and EDS were performed using a Zeiss EVO10 SEM (Pleasanton, CA, USA). Imaging used an acceleration voltage of 20 kV.

## 3. Results and Discussion

**Figure 1:**
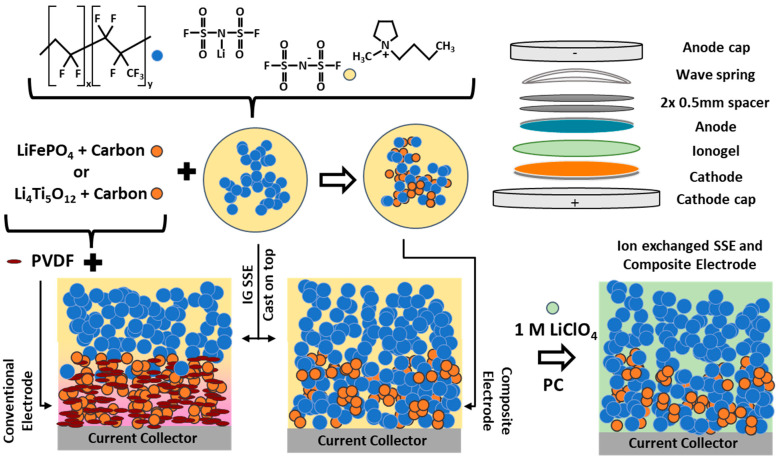
Depiction of ionogel synthesis and how the IG is used to fabricate different versions of anodes/cathodes. From bottom left to right, the depictions shows the conventional cast electrode with deposited IG separator and the composite electrodes with the IG separator layer with subsequent ion exchange. Pink coloring in conventional electrode represents imperfect penetration of IG SSE into electrode layer. Top right shows coin cell construction used to make full cell.

**Figure 2:**
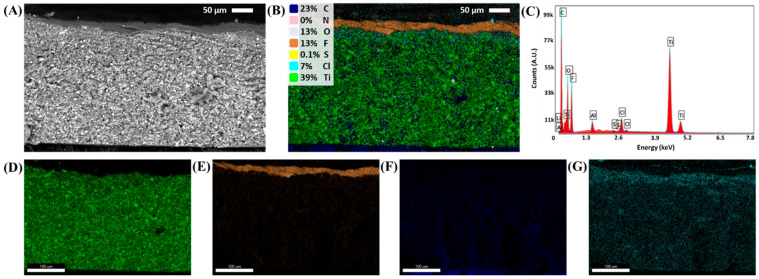
Cross-section imaging of composite LTO with IG separator layer after solvent exchange. (**A**) Backscattering SEM image of electrode–separator layers. (**B**) EDS mapping of elements for same region imaged in (**A**). (**C**) Average spectrum from EDS mapping. (**D**–**G**) Mapping of individual Ti, F, C, and Cl signals, respectively.

As with any battery, utilizing the near-theoretical capacity of the active material is necessary to achieve maximum energy density. To investigate how incorporating an ionogel solid-state electrolyte affects the cycling capacity of LTO, two methods were examined as outlined in Figure 1. The IG SSE was cast onto a conventional LTO electrode, and the SSE precursor material was incorporated into the electrode before deposition. In both cases, a solvent exchange was performed, removing the bulkier counter ions, as demonstrated previously [9]. This was confirmed by taking energy dispersive spectra (EDS) and overlaying each on the corresponding scanning electron microscope (SEM) image of a composite LTO electrode with an IG SSE (Figure 2). The lack of an N and S signal and the presence of Cl correspond to the removal of LiFSI and PYR14TFSI and successful substitution with LiClO_4_, as has been previously demonstrated [8]. The Al signal is likely contamination from the cross-sectioning process. The composite electrode layers were found to be ~250 µm thick and the SSE to be ~20 µm thick by SEM/EDS imaging.

As seen in Figure 2A, the half cell performance of the conventional LTO electrode with an IG SSE achieved an average of 103.9 mAh/g in the first eight cycles at a C/5 rate. The low utilization of only ~61% of the theoretical capacity of LTO is not too surprising given the LTO loading of ~11 mg/cm^2^. Without a sufficient ionic pathway, like there would be in a liquid cell, there is no way to lithiate all of the active material. To combat interfacial contact issues from using an SSE, composite electrodes of active material and SSEs were fabricated. A desirable loading of 10 mg/cm^2^ of active material was targeted to produce electrodes that were not reliant on a small amount of material to produce good electrochemistry and more closely resemble conventionally cast electrodes in commercial Li-ion batteries. Figure 2B shows the early cycling of the composite LTO electrodes cycled versus Li metal in coin cells.

Initially, the high-loading composite electrodes performed quite poorly. The LTO only achieved a maximum of 87.4 mAh/g in early cycles. This is only ~51% of the 170 mAh/g theoretical capacity of LTO and 10% less utilization than the case of using a conventionally cast electrode with the SSE deposited on the surface. To investigate if the issue arose from the LTO or the Li foil, a cell was constructed with a composite lithium titanate and lithium iron phosphate electrode. The early cycling of this cell is shown in Figure 2C. Like the half cell with Li metal, the specific capacity when normalized to the mass of LTO is low, again approaching 50%. While discouraging, unlike a conventional cast electrode with no IG SSE, the amount of ionogel and SSE precursors can be modified in the composite electrode to tune performance. So even though the conventional electrode performed the best from a capacity utilization perspective, the composite electrodes have more tunability to achieve a higher discharge capacity.

In the cycled composite electrodes, the low utilization points to an inability to access the active material. This could be attributed to poor electrical conductivity causing isolated LTO particles to be unreactive, a lack of Li^+^ diffusion through the electrode making lithiation of LTO unattainable, or a complete isolation of active material. To test if the ionic or electrical conductivity needed to be adjusted to improve performance, a series of composite electrodes were fabricated with varying ratios of polymer and conductive carbon. The mass percent of the active material, conductive carbon, and PVDF-HFP used are found in Table 1, along with the ratio of active mass to carbon and active mass to polymer. The motivation for reducing the amount of ionogel or conductive carbon is that too much ionogel may prevent good electrical conductivity, while conversely, too much conducting carbon may prevent rapid Li^+^ diffusion to the LTO.

**Table 1:** Percent loading of active material, conductive carbon, and PVDF-HFP in composite electrodes. Composition 1 was used for LTO electrodes in Figure 3. Values are taken from recorded experimental conditions. Mass percent not totaling 100% is a consequence of not rounding last significant figures.

Composition	Mass Percent Loading			Mass Ratio	
	Active Material	Carbon	PVDF-HFP	Active/Carbon	Active/PVDF-HFP
1	45.29%	19.41%	35.29%	2.33	1.28
2	55.00%	23.57%	21.43%	2.33	2.57
3	62.35%	13.36%	24.29%	4.67	2.57
4	71.30%	10.19%	18.52%	7.00	3.85

**Figure 3:**
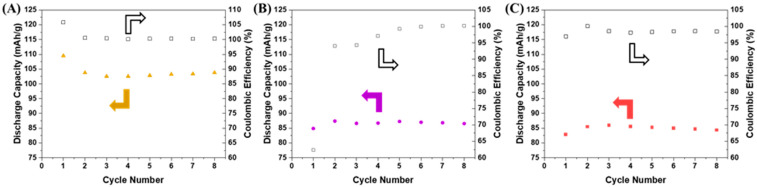
Galvanostatic cycling of half cells using an IG SSE. (**A**) Conventionally cast LTO vs. Li cycling at C/5. (**B**) Composite LTO vs. Li cycling at C/5 rate. (**C**) LTO vs. LFP cycling at C/5 rate. Arrows point to axis associated with data, solid to discharge capacity, and unfilled to Coulombic efficiency.

**Figure 4:**
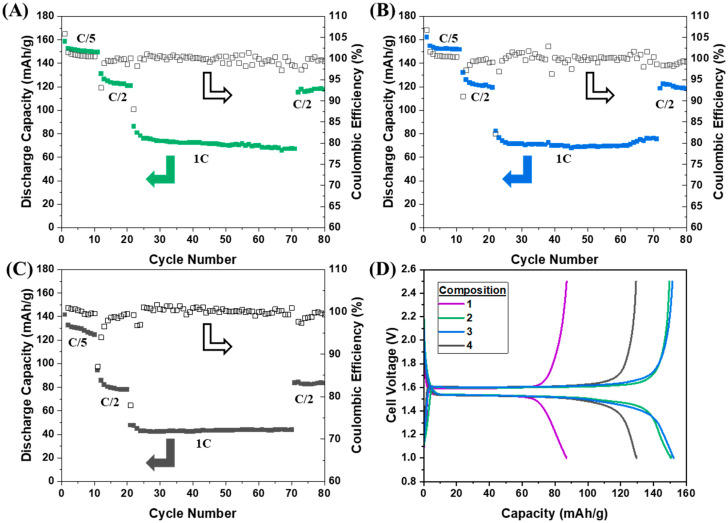
Cycling of LTO composite electrodes vs. Li metal with ionogel SSE at room temperature. The cells were cycled at a rate of C/5 for 10 cycles, C/2 for 10 cycles, 1C for 50 cycles, and then C/2 again for 10 cycles. (**A**) Composition 2. (**B**) Composition 3. (**C**) Composition 4. (**D**) Charge and discharge voltage shown vs. capacity for the 5th cycle of cells in A-C, green, blue, black traces, and composition 1 from the cell in Figure 3A, purple trace. Arrows point to axis associated with data, solid to discharge capacity, and unfilled to Coulombic efficiency.

The capacity and Coulombic efficiencies of cells made with the varying composite ratios are displayed in Figure 4. The cells were cycled at a rate of C/5 for 10 cycles, C/2 for 10 cycles, 1C for 50 cycles, and then C/2 again for 10 cycles, all at room temperature. The corresponding material loadings, current, areal current, and current densities for all of the cycled composite LTO electrodes are provided in Table 2. The differences in active material composition change the areal current/current density between cells with the removal of polymer and carbon, causing an increase in active material loading. Strikingly, all cells performed better than the initial composite LTO electrode presented in Figure 3. All electrodes cycled in Figure 4 had a reduced amount of ionogel compared to the first composition. This is in line with the hypothesis that too much ionogel hinders the electrical conductivity of the electrode, leading to isolated, unreactive LTO. The same amount of carbon was added to compositions 1 and 2, and because composition 2 outperformed the original formulation, this also indicates that the amount of conductive carbon was not the issue in the cell shown in Figure 3A.

While all cells with compositions 2–4 cycled with superior capacity compared to composition 1, their performances were not identical and not all ideal. Compositions 2 and 3 had similar specific capacities at each rate, even with a 50% difference in conductive carbon. At C/5, each on average supplied above 150 mAh/g. While not quite at the theoretical 170 mAh/g of LTO, the active material is carbon-coated (~1–3 wt%), and this coating mass is not accounted for in the specific capacity normalization. Due to this, the electrodes will not achieve 100% LTO theoretical capacity, making 150 mAh/g commendable and closer to the realizable specific capacity. Somewhat surprisingly, these two electrodes are able to support 1C rates corresponding to current densities of ~9 mA/cm^2^, even at room temperature. This achievement would be ideal for a liquid cell and is made even more impressive because these cells are solid state. The voltage profile also maintains the same shape as the smaller current discharges, just with the suppressed voltage expected from the substantial increase in current. Retention of the voltage profile indicates that the electrochemical intercalation mechanism is maintained and that the cell is not relying on pseudocapacitive behavior.

Composition 4 did not achieve as high of capacities at any point when compared to compositions 2 and 3. These latter compositions started with lower capacities in comparison and dipped to even lower utilizations at higher current densities. While this cell was also able to cycle at a 1C rate, the authors believe the lower amount of conductive carbon compared to compositions 2 and 3 makes the electrodes unable to obtain high capacities under higher current loads. Although the utilization was lower, the composite electrode had an average Coulombic efficiency of 99.77% for cycles 2–80. This is slightly larger than for composition 1 (99.68%) but lower than for compositions 2 and 3 (99.88 and 99.86, respectively). As seen in Figure 4D, even with reduced capacities, the voltage profile of compositions 1 and 4 was similar to that of compositions 2 and 3 at a C/5 rate, indicating that iR effects were not responsible for the loss of capacity in compositions 1 or 4. All displayed a flat discharge plateau at ~1.53 V vs. Li.

**Figure 5:**
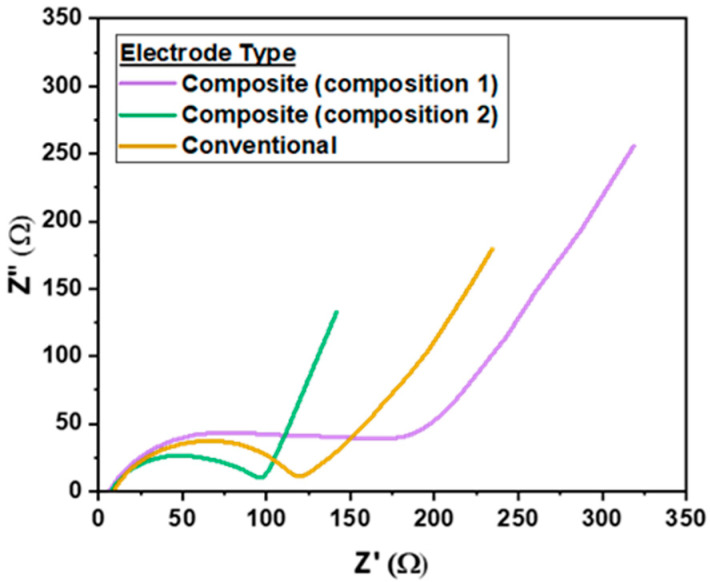
Nyquist plots of EIS measurements on half cells with IG SSE taken from 1 MHz–100 mHz with a 10 mV amplitude and an average of two points per frequency. Composite electrode of composition 2 and then the conventional electrode followed by composition 2 are increasing in impedance.

Electrochemical impedance spectroscopy (EIS) was then used on selected cells to help explain the galvanostatic cycling performance differences observed. In the Nyquist plots seen in Figure 5, there is clearly an impact on cell resistance and capacitance depending on the amount of IG SSE included (or not) in the LTO electrode formulation. From an impedance perspective, the worst cell is the composition 1 LTO electrode, which has the largest charge transfer resistance and capacitance, which is surmised from the theoretical Z’ intercept of the right portion of the semicircle and the maximum Z” of the semicircle, respectively [24,25]. The elongated semicircle feature in this curve likely arises from multiple electrochemical interfaces being produced from the high ionogel content in the composite electrode. The EIS of the conventional LTO electrode in a Li half cell with an IG SSE had a similar maximum capacitance but lower charge transfer resistance than the cell with a composition 1 anode. The composition 2 LTO in comparison had the best EIS properties, with lower charge transfer resistance and decreased capacitance. The overall lower impedance of composite 2 is likely responsible for creating the conditions the cell needs to have near-theoretical capacity utilization at C/5, where composition 1 and the conventional electrode are hindered by poor ion and electron transport.

**Figure 6:**
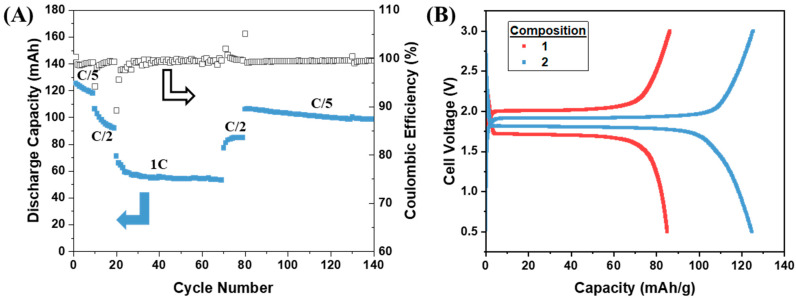
Composition 2 LTO electrode vs. composite LFP with ionogel SSE. (**A**) Discharge capacity and Coulombic efficiency shown for cycles cycled at a rate of C/5 for 10 cycles, C/2 for 10 cycles, 1C for 50 cycles, and then C/2 again for 10 cycles and C/5 for 60 cycles. Specific capacity normalized by mass of LTO. Arrows point to axis associated with data, solid to discharge capacity, and unfilled to Coulombic efficiency. (**B**) Voltage profile of cell in Figure 3B [composition 1] plotted in red shown against voltage profile of cell from (**A**) [composition 2] plotted in blue.

Following the formulation of composition 2, a full solid-state cell with a composite LTO and LFP electrode was constructed each with active loadings ~10 mg/cm^2^. This composition was chosen because of the capacity utilization, Coulombic efficiency, and the longer voltage plateau before polarization at the end of discharge. This loading is also higher than typical loadings of solid-state-type cells reported in the literature (Table 3) [20,26,27,28,29,30,31]. The authors do not claim that this is an extensive or exhaustive list of works, but it represents common loadings used in cell fabrication. As seen in Figure 6, the LTO||LFP cell was cycled in a similar fashion to the LTO||Li half cells. However, the maximum capacity at the reach rate was diminished compared to the Li half cells. Therefore, LFP was the limiting factor in the cell capacity. Even with lower capacity, the cell maintained a stable capacity at a 1C rate and showed good capacity recovery when cycled at C/2 again. The capacity retention was also above 80% after 140 cycles, indicating that the high-rate cycling did not significantly degrade the composite electrodes. The Coulombic efficiency remained high throughout cycling with an average of 99.42%. Larger changes in Coulombic efficiency were seen when then the cell underwent changes in current load, similar to the half cells.

Like the composition-varying process performed for the LTO electrodes, we expect a similar investigation would be necessary to optimize the composite formulation for the LFP electrode and the n/p ratio for the electrodes. However, this fully polymeric solid-state cell displayed over a 46% increase in capacity compared to the initial attempt shown in Figure 3B. Not only was the capacity improved but the discharge voltage was increased by ~100 mV throughout the discharge plateau and decreased by 90 mV during charging. Using this new composition for the electrodes, therefore, improves energy efficiency while simultaneously improving energy density. This improvement is possible exclusively through adjusting the electrode composition.

## 4. Conclusions

Ionogels enable the formation of solid-state electrolytes and composite electrodes for polymer-based solid-state batteries operating at room temperature. Here, the development of composite lithium titanate electrodes was demonstrated. The ratio of conductive carbon and ionogel was found to be essential to improving rate capability and the capacity utilization of the LTO. By varying the composition, the capacity of the LTO composite electrodes was improved by 70 mAh/g and shown to cycle stably at a 1C rate. The high-rate capabilities of these high-loading composite electrodes with SSEs show that all solid-state systems are worth continuing to develop to compete with liquid cells. The optimized LTO anode was cycled against a composite LFP cathode and also shown to support high rates in a solid-state cell. The discharge voltage and energy efficiency were both improved by changing the ionogel content of the electrodes. Further optimization of the cathode could enable even higher capacity utilization at rates above 1C. This includes introducing unique architecture and advanced manufacturing techniques, like 3D printing, for custom cell designs.

## Figures and Tables

**Table 2:** Electrode capacities and the corresponding rates used during cycling given in mA, mA/cm^2^, and A/g.

Composition	Active Material (mg/cm^2^)	Capacity (mAh)	Rate (mA)			Rate (mA/cm^2^)			Rate (A/g)		
			C/5	C/2	C/1	C/5	C/2	C/1	C/5	C/2	C/1
1	7.76	2.49	0.50	1.24	2.49	1.32	3.30	6.60	0.034	0.085	0.17
2	10.52	3.37	0.67	1.69	3.37	1.79	4.47	8.94	0.034	0.085	0.17
3	10.55	3.38	0.68	1.69	3.38	1.79	4.48	8.97	0.034	0.085	0.17
4	13.76	4.41	0.88	2.21	4.41	2.34	5.85	11.70	0.034	0.085	0.17

**Table 3:** Reported active material loadings in electrodes for solid-state batteries.

Electrolyte	Active Material	Loading mg/cm^2^	Reference
PU-SN-LiTFSI	LFP||Li	1	[26]
PVDF-HFP-LiTFSI-NMP	LFP||Li	1.5	[19]
PVDF-HFP-LiMNT-PEG	LFP||Li	1.2	[30]
LLZO-polymerized DOL gel bilayer	SPAN||Li	5.2	[20]
LLZO-plastic crystal electrolyte (LiTFSI-SN)	LFP||Li	4.88	[28]
GO-PEO	LFP||Li	2.5	[29]
PEO-LiTFSI	LTO||Li	3	[31]
Solvent Exchanged PVDF-HFP	LFP||LTO	10.5	This Work

Polyurethane (PU), lithium lanthanum zirconium oxide (LLZO), sulfurized polyacrylonitrile (SPAN), Dioxolane (DOL), graphene oxide (GO), polyethyleneoxide (PEO), lithium montmorillonite (LiMNT), polyehtlyne glycol (PEG), succinonitrile (SN).

## Data Availability

The original contributions presented in the study are included in the article, further inquiries can be directed to the corresponding author.
